# Effects of Synthesis Conditions on the Formation of Si-Substituted Alpha Tricalcium Phosphates

**DOI:** 10.3390/ijms21239164

**Published:** 2020-12-01

**Authors:** Katarzyna Szurkowska, Łukasz Szeleszczuk, Joanna Kolmas

**Affiliations:** 1 Chair of Analytical Chemistry and Biomaterials, Department of Analytical Chemistry, Faculty of Pharmacy, Medical University of Warsaw, ul. Banacha 1, 02-097 Warsaw, Poland; katarzyna.szurkowska@wum.edu.pl; 2 Chair of Physical Pharmacy, Department of Physical Chemistry, Faculty of Pharmacy, Medical University of Warsaw, ul. Banacha 1, 02-097 Warsaw, Poland; lukasz.szeleszczuk@wum.edu.pl

**Keywords:** α-TCP, calcium phosphates, bioceramics, biomedical applications, physico–chemical properties

## Abstract

Powders of α-TCP containing various amounts of silicon were synthesized by two different methods: Wet chemical precipitation and solid-state synthesis. The obtained powders were then physico–chemically studied using different methods: Scanning and transmission electron microscopy (TEM and SEM), energy-dispersive X-ray spectroscopy (EDS), powder X-ray diffractometry (PXRD), infrared and Raman spectroscopies (FT-IR and R), and solid-state nuclear magnetic resonance (ssNMR). The study showed that the method of synthesis affects the morphology of the obtained particles, the homogeneity of crystalline phase and the efficiency of Si substitution. Solid-state synthesis leads to particles with a low tendency to agglomerate compared to the precipitation method. However, the powders obtained by the solid-state method are less homogeneous and contain a significant amount of other crystalline phase, silicocarnotite (up to 7.33%). Moreover, the microcrystals from this method are more disordered. This might be caused by more efficient substitution of silicate ions: The silicon content of the samples obtained by the solid-state method is almost equal to the nominal values.

## 1. Introduction

Calcium phosphates (CaPs) have received a great deal of attention as potential biomaterials for bone and teeth applications due to their chemical similarity to biological apatite, the main inorganic constituent of human mineralized tissues [1,2,3]. So far, the most thoroughly studied compound has been hydroxyapatite (HA), Ca_10_(PO_4_)_6_(OH)_2_, and its ionic-substituted forms [4,5,6,7]. Despite its high biocompatibility, it has some limitations. The main disadvantage of HA as an implantable material is insufficient solubility resulting in poor in vivo resorption. The use of other CaPs with greater solubility allows for enhanced resorption, and thus increases the bioactivity of the material [8,9]. Therefore, there is a great interest in tricalcium phosphate (TCP, Ca_3_(PO_4_)_2_) as a bone replacement material [10,11].

TCP with Ca/P molar ratio 1.5 occurs in different polymorphic forms: The low temperature beta-TCP (β-TCP) and two high-temperature varieties, alpha- and alpha α’-TCP (α- and α’-TCP) [12,13,14,15]. The latter, α’-TCP, is stable only at temperatures >1430 °C, therefore, practically, it cannot be used as biomaterial. β-TCP is formed when a CaP with Ca/P ratio between 1.5 and 1.67 is sintered in the range 700 to 1125 °C. β-TCP is mainly used in biphasic calcium phosphate (BCP) biomaterials, where it is combined with HA in various weight ratios in order to prepare biodegradable granules and blocks [14].

In contrast, high-temperature α-TCP is synthesized during heat treatment exceeding 1125 °C [12,13]. When compared with β-TCP, α-TCP is less dense, more soluble and exhibits higher reactivity with water which may lead to the formation of calcium-deficient HA (Equation (1) [15]):

3 α-Ca_3_(PO_4_)_2_ + H_2_O → Ca_9_(PO_4_)_5_(HPO_4_)OH. (1)

α-TCP is mainly used in the form of a powder to produce calcium phosphate bone cements (CPCs), to fill bone defects and eventually become resorbed and replaced by newly formed osseous tissue.

Unfortunately, α-TCP is metastable at room temperature. In order to avoid the formation of other phases, such as β-TCP, it should be rapidly quenched during synthesis to room temperature. Recently, it has been found that several dopants may affect the α-β transition [16,17,18]. One of the factors stabilizing α-TCP at room temperature is silicon. Recent research proved that the introduction of Si into the α-TCP structure, enables easier production at lower temperatures [17,18].

In addition to the stabilizing effect on the crystalline phase, silicon stimulates the bioactivity of the material and potentially improves the clinical therapeutic effect of the bone repair [19].

According to the research of Reid et al. [17] the limit of Si substitution to α-TCP is approx. 1.8 wt%. As a result, saturated material with general formula (Ca_3_(P_0.9_Si_0.1_O_3.95_)_2_ may be obtained. 

Silicon substitution in CaPs has been widely studied since the ground-breaking research by Carlisle and Schwarz on the essential role of silicon in bone and connective tissue development [20,21]. It has been shown that silicate ions stimulate osteoblast activity and differentiation as well as collagen type I synthesis [22]. In vitro studies have shown the dose-dependent effect on osteoclasts [23]. Moreover, silicates released from biomaterials not only stimulate osteogenesis but also angiogenesis [24]. 

It should be noted, that in medical practice, four commercial preparations containing Si-doped CaPs are used: Actifuse (Apatech Ltd., Elstree, UK) and Inductigraft (Baxter Healthcare, Deerfield, IL, USA) consisting of a single phase Si-HA, BoneMedik-S (Meta Biomed Co. Ltd., Cheongju, Korea) consisting of Si-HA derived from coral, and Skelite (Millenium Biologix, Kingston, ON, Canada), a combination of 67% Si-αTCP and 33% HA [25].

Si-substituted CaPs (mainly Si-HA) have been widely studied over the last few years and the research continues [26,27,28]. It should be emphasized that the beneficial effects of Si-doping in α-TCP is still poorly understood and requires more examination.

The purpose of the work is a deeper understanding of the possibilities of α-TCP substitution with silicate ions. The research summarizes the two most popular methods of synthesis of silicon-substituted α-TCP (Si-αTCP): Precipitation [29] and solid-state synthesis [30]. Various synthesis conditions were evaluated in terms of phase purity, substitution efficiency, and morphology of the obtained powders.

## 2. Results and Discussion

### 2.1. Scanning and Transmission Electron Microscopy

**Figure 1:**
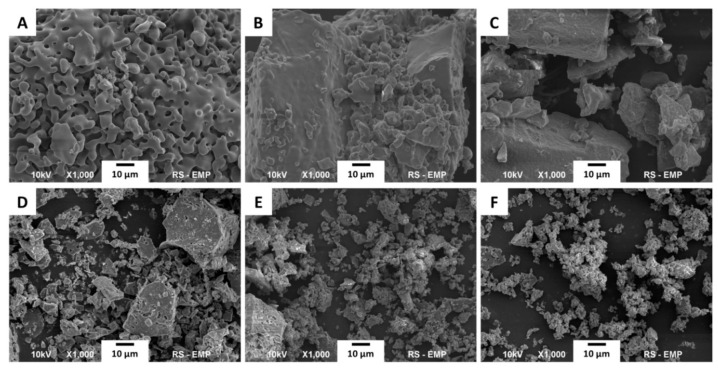
SEM microphotographs of the samples: 09Si-aTCP (**A**), 18Si-aTCP (**B**), 37Si-aTCP (**C**), 86 09Si-aTCP**s** (**D**), 18Si-aTCP**s** (**E**), and 37Si-aTCP**s** (**F**).

Figure 1 illustrates the agglomerates of particles assembled from a number of crystals. The SEM images revealed that the powders obtained by various synthesis methods exhibit significant differences in morphology and grain size. The samples obtained by the precipitation method (x-aTCP samples, where x is the nominal amount of Si in the samples and aTCP stands for α-tricalcium phosphate) consist of large particles with a size of several micrometers (2−10 μm). In the 18Si-aTCP and 37Si-aTCP samples, the particles are fused together in such a way that they form compact structures. The 09Si-aTCP sample is more porous and exhibits a typical morphology for sintered powders [31].

The samples obtained via the solid-state route are characterized by finer particles with a size below 1 μm. They show a much lower tendency to agglomerate and to form compact, fused structures. It may be assumed that in the solid-state method of synthesis the addition of silicon inhibits particle growth and causes nucleation of more particles with lower volume.

The SEM results are consistent with TEM images presented in Figure S1 in Supplementary Materials. Due to the considerable thickness and size of a single particle, its analysis was impossible for TEM microscopy. However, in all the samples it can be observed that the crystals are fused together and form spherically shaped molds, reaching a size of up to tens of microns.

### 2.2. Powder X-ray Diffractometry

**Figure 2:**
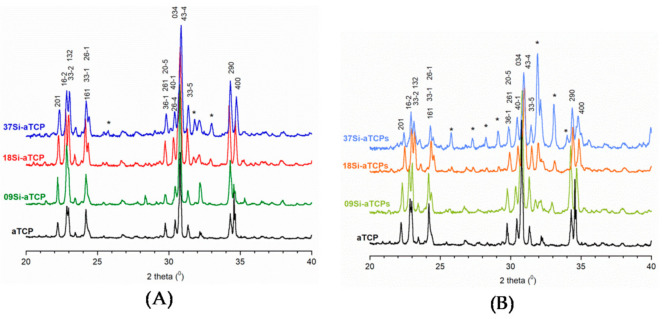
Powder X-ray diffractometry (PXRD) diffractograms of the x-aTCP (**A**) and x-aTCPs (**B**) samples (*—silicocarnotite).

Diffractograms of all the obtained materials are compared in Figure 2A,B. Only for the 09Si-aTCP sample does it seem to reveal almost exclusively α-TCP reflections (JCPDS #9-348 and Figure S2 in Supplementary Materials). For all the samples the peaks from α-TCP are dominant, conspicuous and well-separated. However, it should be noted that the peaks on the diffractograms of the samples synthesized by the solid-state method are slightly more blurred and less resolved (see Figure 2A,B, especially (16–2), (290) and (400) reflections. As observed, the peaks are broader as the content of silicon increases (see Figure 2B).

Moreover, a significant change in the position of all peaks and their shift towards higher 2 theta values can be easily observed in the 37Si-aTCP, 18Si-aTCPs, and 37Si-aTCPs diffractograms (see Table S1 in Supplementary Materials). We can therefore assume that silicates affect the α-TCP structure and that their introduction was at least partly successful [32].

Introducing silicates, especially during the synthesis via the solid-state route, leads to extra peaks on diffractograms, which are attributable to the silicocarnotite phase [33,34]. Silicocarnotite (SC) with the general formula Ca_5_(PO_4_)_2_SiO_4_ is an inorganic material which can be formed during the calcination of calcium phosphates with silicon-containing compounds [34]. The quantitative phase analysis estimates are presented in Table 1. Samples synthesized by the precipitation method contain the silicocarnotite phase in an amount not exceeding 3.1%. In turn, in the samples from the solid-state method, the amount of silicocarnotite phase increases with the nominal amount of silicon introduced (in the 37Si-aTCPs sample the silicocarnotite content is as high as 7.33%). For comparison, the PXRD diffractograms of the “raw”, unsintered powders obtained by precipitation method are presented in Figure S3 (in Supplementary Materials). As expected, the “raw” samples show the structure of nanocrystalline apatite (see Table S2 in Supplementary Materials). As the nominal amount of silicon increases, the peaks are broader and less separated, which indicates a higher disorder of the apatitic structure. The introduction of silicon ions causes a decrease in the parameter *a* and an increase in the parameter *c*, that it is in a great accordance with the literature data [25,28].

The lattice parameters of the obtained α-TCP phases in each sample are shown in Table 1. The greatest increases of lattice are observed along *b* length. This is in accordance with previous research [32,35,36]. The unit cell lengths along *a* and *c* axes as well as *β* angle, change with the increase in silicon, but in an irregular manner. It should be noted that *a* and *c* parameters for the 18Si-aTCPs and 37Si-aTCPs samples containing the largest amount of silicocarnotite (5.04% and 7.33%, respectively) decrease, whilst according to the literature, they should increase [32,35,36]. The reason for this may be the partial incorporation of silicates into the silicocarnotite phase.

### 2.3. Quantitative Analysis of Silicon

The results of measurements of Si content are summarized in Table 1. The obtained values are close to the nominal values (see Section 3.1); in the samples from solid-state synthesis, sometimes even exceeding them. However, it is worth noting that the calculated values refer to the whole sample, i.e., to the sum of the Si-α-TCP and the silicocarnotite phases. Taking into account the actual composition of the samples, the nominal wt% of Si in the obtained sample is presented in brackets (Table 1). With the precipitation method, a lower efficiency of Si introduction into the sample can be observed. This is most likely due to the losses resulting from washing the precipitates with distilled water. In turn, the silicon content of the samples obtained by the solid-state method is almost equal to the nominal values.

### 2.4. Vibrational Spectroscopy

**Figure 3:**
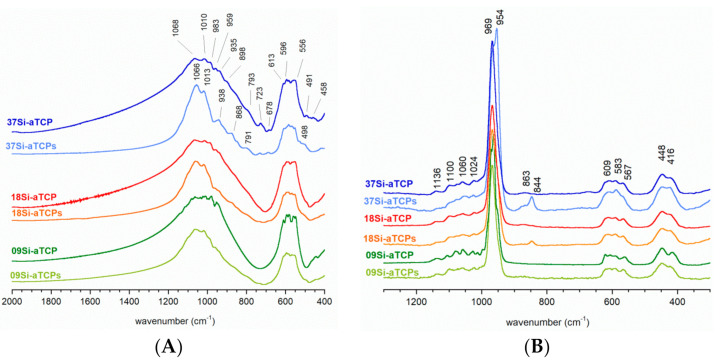
FTIR (**A**) and Raman (**B**) studies.

The FT-IR spectra of the obtained samples (Figure 3A and Table 2) show the bands characteristic of orthophosphate ions at 1068–1066 cm^−1^, 1013–1010 cm^−1^, 959 cm^−1^, 613–556 cm^−1^, and 458 cm^−1^ assignable to ν_3_(PO_4_), ν_1_(PO_4_), ν_4_(PO_4_) and ν_2_(PO_4_), respectively. The detailed study on vibrational spectroscopy of tricalcium phosphates provided by Jillavenkatesa and Condrate [37] showed that the FT-IR spectrum of αTCP is very complex and may theoretically contain over two hundred phosphate bands: ν_1_—24 distinct bands, ν_2_—48 bands, ν_3_, and ν_4_—72 bands each. Of course, not all theoretical bands have to be visible in the spectrum. This can be due to their low intensity or insufficient resolution of the spectrometer.

The bands observed in the obtained spectra are very poorly resolved giving broad lines with only slight splitting on the top. Particularly wide bands appear in the spectra of the samples synthesized by the precipitation method (09Si-aTCP, 18Si-aTCP, and 37Si-aTCP). It should be noted that these spectra are similar to the standard α-TCP [12,13,37], and are in accordance with PXRD results. In turn, the ν_1_ + ν_3_ phosphate bands in the spectra of samples 09Si-aTCPs, 18Si-aTCPs and 37Si-aTCPs are significantly narrower. What is more, the ν_1_ + ν_3_/ ν_4_ intensity ratio is visibly higher than in the spectra of the precipitated samples and may suggest the presence of another orthophosphate phase [38]. The positions of the bands could indicate the presence of hydroxyapatite or oxyhydroxyapatite as an impurity of the samples [39,40]. However, it must be noted that in the obtained spectra the band of the structural hydroxyl group at 3570 cm^−1^ characteristic for HA was not found, even in the spectrum of the 37Si-aTCP sample (see Supplementary Materials, Figure S2). This is in agreement with the PXRD results, which revealed the addition of only a silicocarnotite phase.

The infrared bands of silicates are not very intense in the spectra of the synthesized samples and unfortunately appear in the same ranges as the orthophosphates. However, some shoulders can be easily detected: 983 and 898 cm^−1^ ν_3_(SiO_4_), 491-498 cm^−1^ ν_2_(SiO_4_), and 935 cm^−1^ (Si-OH) [41,42]. In the spectrum of the 37Si-aTCP sample, two new additional bands appeared at 868 and 791 cm^−1^ originating from Si-O-Si vibrations, which may be related to the formation of an amorphous silica phase [41].

Considering the complexity of the FT-IR spectra of the obtained samples, the Raman spectra were also provided (see Figure 3B and Table 2). The typical spectra of α-TCP sample were obtained, with the most dominant bands at 969 and 954 cm^−1^ originating from ν_1_ phosphate vibrations. Slight differences in resolution of the phosphate bands in the regions of 1136–1024 cm^−1^ (ν_3_), 609–567 cm^−1^, (ν_4_) and 448–416 cm^−1^ (ν_2_) can be detected. Moreover, in the 18Si-aTCPs and 37Si-aTCPs spectra, additional bands in the 863–844 cm^−1^ occur. According to [33,42] this may be assigned to silicates in silicocarnotite. The absence of the bands typical for silica at approximately 800 cm^−1^ indicates its low concentration in the samples.

### 2.5. Solid-State ^31^P NMR MAS Spectroscopy

Phosphorus-31 resonance spectra were collected using BD and CP techniques, giving the signals from all ^31^P nuclei in the sample and from the ^31^P nuclei located close to protons, respectively. As expected, due to the lack of protons, all the ^31^P CP MAS NMR spectra reveal no signals (data not shown). It may be concluded that the samples do not contain residual protons from water or from hydroxyl groups [43]. This is in an accordance with the FT-IR results, where no bands were detected from water or from structural OH groups.

**Figure 4:**
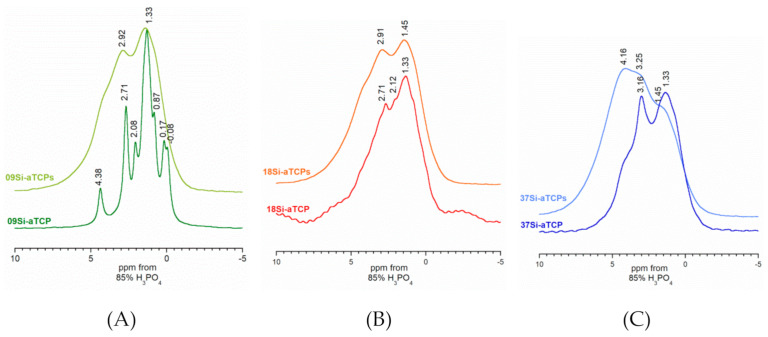
^31^P BD NMR spectra of the obtained powders: 09Si-aTCP and 09SiaTCPs (**A**), 18Si-aTCP and 18Si-aTCPs (**B**), 37Si-aTCP and 37Si-aTCPs (**C**).

The ^31^P BD MAS NMR spectra look totally different (see Figure 4A–C). The spectrum of the 09Si-aTCP sample most closely resembles the ^31^P BD spectrum of standard, pure α-TCP [13,32,44]. The signals are quite narrow and well resolved. As the Si content in the samples increases, the lines become significantly broader and difficult to detect (see Figure 4C) suggesting a structural disorder in Si-substituted samples. What is more, all the spectra of the samples synthesized by the solid-state method contain only two readily delineable signals. Therefore, it can be assumed that the structural stability is not only affected by the amount of introduced silicon, but also by the method of synthesis: The solid-state method seems to be favourable for the formation of the disordered Si-αTCP structure. This may be due to the presence of a significant amount of silicocarnotite in the sample which, according to the literature gives a rather broad resonance line at approximately 4.6 ppm [45].

## 3. Materials and Methods

### 3.1. Preparation of Samples

Two series of silicon-substituted alpha tricalcium phosphates with three different concentrations of silicon were synthesized via wet precipitation and solid-state routes. On the basis of the chemical formula of Si-αTCP; Ca_3_P_2-x_Si_x_O_8-0.5x_, the quantity of reagents was calculated assuming a Ca/(P+Si) molar ratio of 1.50 and that one phosphate ion would be substituted for one silicate ion. All reagents were purchased from Sigma-Aldrich (St. Louis, MO, USA).

In the first step of the precipitation method, 50 mL of diammonium hydrogen phosphate [(NH_4_)_2_HPO_4_] and silicon acetate [Si(OCOCH_3_)_4_] aqueous solution were added dropwise to 400 mL of calcium nitrate [Ca(NO_3_)_2_∙4 H_2_O] solution. The pH of the mixture was set at 10 by the addition of ammonia. The reaction suspension was stirred for 2 h at room temperature and then left without stirring for 24 h to age. The resulting precipitate was centrifuged and rinsed with distilled water several times in order to remove soluble products. In the second step, the obtained powder was dried at room temperature, then sintered in the muffle furnace for the phase transition into Si-αTCP. The following temperature programme was used: Heating up to 1250 °C with a heating rate of 3 °C/min., then sintering at 1250 °C for 7 h and cooling to 300 °C with a cooling rate of 2 °C/min. When the temperature reached 300 °C the samples were left to cool down in the furnace without quenching.

For the solid-state route, calcium carbonate [CaCO_3_], diammonium hydrogen phosphate [(NH_4_)_2_HPO_4_] and finely dispersed fumed silica [SiO_2_] were used as calcium, phosphate and silicon sources, respectively. The reactants were carefully mixed together in a ball mill for 30 min, pressed into pellets using a hydraulic press and then sintered with the same temperature programme as for precipitated powders in the first method. After the thermal treatment, all the samples were crushed in an agate mortar and subjected to further examination.

For comparison, the unsubstituted samples were synthesized by both methods. The obtained samples are listed in Table 3.

### 3.2. Sample Sharacterization

Microstructural features of the obtained powders were studied using microscopy: Transmission (TEM) and scanning (SEM) electron microscopy. In order to observe shapes of microparticles, TEM measurements were performed with a JEOL JEM-1400 microscope (Tokyo, Japan) with an accelerating voltage of 80 kV. Prior to analysis, the samples were suspended in ethanol and then dropped onto a copper grid covered with Formvar. To analyse the surface morphology of the particles and their tendency to agglomerate, a SEM microscope JSM 6390 LV JEOL (Tokyo, Japan) was used. To carry out SEM analysis, the surface layer of the samples were gold-coated in a vacuum chamber.

SEM microscopy ZEISS AURIGA 60 (Oberkochen, Germany) equipped with XFlash 6|10 Bruker (Billerica, MA, USA) EDS (energy dispersive spectrometer) was also used for quantitative analysis of silicon. Silicon content was measured at 20 kV accelerating voltage from 10 spots and then averaged. Powder X-ray Diffractometry (PXRD) was used to investigate the phase compositions of all the samples. Data were collected using Bruker DX8 Discover (Billerica, MA, USA) diffractometer with CuKα radiation (λ = 1.54 Å); over the 2 theta range of 20°–70° with a step size of 0.024°, a step time of 4 s (s) and a locked, coupled (theta-theta) geometry. The lattice parameters *a*, *b,* and *c* and *β* angle of the unit cells were determined with the BIOVIA Materials Studio 8.0 (San Diego, CA, USA) Reflex module software [46], using Rietveld refinement based on analytical profile functions and least-squares algorithms in order to fit a theoretical diffractogram to experimental lines.

Quantitative Phase Analysis (QPA) was also performed using BIOVIA Materials Studio 8.0. The QPA type and convergence quality were set to full refinement and ultrafine level respectively, using the entire range of 2 theta angles to compare with the experimental PXRD pattern (10°–90°). The Rietveld method, applied for quantification of phases, is a type of standardless QPA method using crystal structures of the main components and experimental powder diffraction patterns of the mixture as input. In this case, the simulated diffraction pattern of the mixture is represented as the superposition of powder diffraction patterns simulated from the refined crystal structures of the component phases. The crystal structures of α-TCP (refcode: 2106194) and silicocarnotite (refcode: 9011950) were obtained from Crystallography Open Database [47].

FT-IR spectra were obtained using a Perkin Elmer Spectrum 1000 spectrometer (Waltham, MA, USA). Data were collected within 30 scans, using the KBr pellets transmission technique in the frequency range of 4000–400 cm^−1^ and 2 cm^−1^ spectral resolution.

The Raman spectroscopy measurements were performed using iRaman 532 (B&W Tek, Newark, DE, USA) spectrometer. The conditions were 20× with a laser wavelength excitation of 532 nm. The spectra were recorded from 150 to 4000 cm^−1^ at room temperature.

All of the obtained spectra (Raman and FT-IR) were processed using GRAMS/AI 8.0 (Thermo Fisher Scientific, Waltham, MA, USA) and KaleidaGraph 3.5 (Synergy Software, Reading, PA, USA) software.

Solid-state ^31^P one-pulse Bloch decay (BD) and ^1^H→^31^P cross-polarization (CP) NMR experiments were performed at room temperature on a Bruker WB 400 spectrometer (Billerica, MA, USA) using a Bruker 4 mm probe. The samples were spun in ZrO2 rotors with MAS frequency of 7 kHz. The ^31^P chemical shifts were referenced to external 85% orthophosphoric acid. ^31^P BD MAS NMR spectra were collected using a 90 s recycle delay and a 90° pulse of 2.75 µs, while ^31^P CP MAS NMR were performed using a 90° pulse of 2.85 µs, a recycle delay of 10 s and a 2 ms contact time. The number of scans was set to 32. The NutsPro 2D (AcornNMR, Livermore, CA, USA) software package was used for all line fitting procedures.

## 4. Conclusions

In this study, two series of the Si-aTCP powders were synthesized by two different methods: Chemical precipitation and the solid-state route. Powders obtained by solid-state synthesis are characterized by much finer particles with a low tendency to agglomerate compared to powders from the precipitation method. In turn the xSi-aTCP samples are more homogeneous, they contain almost exclusively α-TCP; the phase of silicocarnotite is up to 3.06 wt%. The efficiency of introducing silicon into the samples is higher for solid-state synthesis. However, silicon is located not only in α-TCP, but also in silicocarnotite, which content is up to 7.33 wt% (for the 37Si-aTCPs sample). The results of this study could pave the way to finding the optimal, efficient method to obtain Si-enriched α-TCP for potential applications in materials science. Future research will focus on the evaluation of the biological activity of the obtained powders and on mechanical tests.

## Figures and Tables

**Table 1:** Chemical compositions of the obtained powders (quantitative phase analysis, Si wt% contents) and lattice parameters from PXRD studies.

Sample	Composition [wt%]		Si Content [wt%]	Lattice Parameters				
	Si-α-TCP	SC		*a* [Å]	*b* [Å]	*c* [Å]	*β* [⁰]
**09Si-aTCP**	97.15	2.85	0.85 (1.05 *)	12.909	27.268	15.211	125.80
**18Si-aTCP**	97.62	2.37	1.59 (1.91 *)	12.875	27.341	15.239	125.76
**37Si-aTCP**	96.94	3.06	2.44 (3.74 *)	12.900	27.356	15.224	125.66
**09Si-aTCPs**	96.60	3.40	1.06 (1.07 *)	12.892	27.345	15.235	125.73
**18Si-aTCPs**	94.96	5.04	2.06 (2.11 *)	12.911	27.564	15.189	125.77
**37Si-aTCPs**	92.64	7.33	3.78 (3.78 *)	12.874	27.202	15.282	125.80

* Nominal wt% of Si in the obtained sample.

**Table 2:** Main bands and their characteristic wavenumbers in FT-IR and Raman spectroscopy (α-TCP and SC (silicocarnotite)).

IR (cm^−1^)	Raman (cm^−1^)	Normal Modes (Assignments)
458	448	symmetric P-O bending υ_2_ (α-TCP and SC)
491–498		υ_2_ SiO_4_^4−^
556 596 613	567 583 609	anti-symmetric P-O bending υ_4_ (α-TCP and SC)
678 723		υ_1_ SiO_4_^4−^
791–793		silica (SiO_2_)
868 898	844	υ_3_ SiO_4_^4−^ silica (SiO_2_)
935–938 959	969–954	symmetric P–O stretching, υ_1_ (α-TCP and SC) symmetric P–O stretching, υ_1_ (α-TCP)
1010–1013 1066–1068	1024 1060 1100 1136	anti-symmetric P–O stretching, υ_3_ (α-TCP and SC)

**Table 3:** Designation and nominal composition of Si-αTCP powders.

Sample	Synthesis Method	Silicon Source	Expected Chemical Formula	Expected Si Content [wt%]
**09Si-aTCP**	precipitation	silicon acetate	Ca_3_(P_0.95_Si_0.05_O_3.975_)_2_	0.9
**18Si-aTCP**			Ca_3_(P_0.9_Si_0.1_O_3.95_)_2_	1.8
**37Si-aTCP**			Ca_3_(P_0.8_Si_0.2_O_3.9_)_2_	3.7
**09Si-aTCPs**	solid state	fumed silica	Ca_3_(P_0.95_Si_0.05_O_3.975_)_2_	0.9
**18Si-aTCPs**			Ca_3_(P_0.9_Si_0.1_O_3.95_)_2_	1.8
**37Si-aTCPs**			Ca_3_(P_0.8_Si_0.2_O_3.9_)_2_	3.7

## Supplementary Materials

Supplementary materials can be found at https://www.mdpi.com/1422-0067/21/23/9164/s1. Figure S1. TEM images of 09Si-aTCP (A), 18Si-aTCP (B), 37-aTCP (C), 09Si-aTCPs (D), 18Si-aTCPs (E) and 37Si-aTCPs (F). Figure S2. PXRD diffractograms of the aTCP and aTCPs samples. Figure S3: PXRD diffractograms of the raw, unsintered samples obtained by precipitation method. Figure S4. FTIR spectra in 4000–400 cm^−1^ range. Table S1. Main peak positions in PXRD diffractograms. Table S2. PXRD Data.

## References

[1] Vallet-Regi M., Gonzalez-Calbet J.M. (2004). Calcium phosphates as substitution of bone tissues. Prog. Solid State Chem..

[2] Best S.M., Porter A.E., Thian E.S., Huang J. (2008). Bioceramics: Past, present and for the future. J. Eur. Ceram. Soc..

[3] Elliot J. (1994). Structure and Chemistry of the Apatites and Other Calcium Orthophosphates.

[4] Kalita S.J., Bhardwaj A., Bhatt H.A. (2007). Nanocrystalline calcium phosphate ceramics in biomedical engineering. Mater. Sci. Eng. C.

[5] Šupová M. (2015). Substituted hydroxyapatites for biomedical applications: A review. Ceram. Int..

[6] Shepherd J.H., Shepherd D.V., Best S.M. (2012). Substituted hydroxyapatites for bone repair. J. Mater. Sci. Mater. Med..

[7] Uskoković V. (2020). Ion-doped hydroxyapatite: An impasse or the road to follow?. Ceram. Int..

[8] LeGeros R.Z. (1993). Biodegradation and bioresorption of calcium phosphate ceramics. Clin. Mater..

[9] Tadic D., Epple M. (2004). A thorough physicochemical characterisation of 14 calcium phosphate-based bone substitution materials in comparison to natural bone. Biomaterials.

[10] Laskus A., Kolmas J. (2017). Ionic substitutions in non-apatitic calcium phosphates. Int. J. Mol. Sci..

[11] Uskoković V., Uskoković D.P. (2011). Nanosized hydroxyapatite and other calcium phosphates: Chemistry of formation and application as drug and gene delivery agents. J. Biomed. Mater. Res. B Appl. Biomater..

[12] Carrodeguas R.G., De Aza S. (2011). α-Tricalcium phosphate: Synthesis, properties and biomedical applications. Acta Biomater..

[13] Kolmas J., Kaflak A., Zima A., Slósarczyk A. (2015). Alpha-tricalcium phosphate synthesized by two different routes: Structural and spectroscopic characterization. Ceram. Int..

[14] LeGeros R.Z., Lin S., Rohanizadeh R., Mijares D., LeGeros J.P. (2003). Biphasic calcium phosphate bioceramics: Preparation, properties and applications. J. Mater. Sci. Mater. Med..

[15] Dorozhkin S.V. (2009). Calcium orthophosphate cements and concretes. Materials.

[16] Frasnelli M., Sglavo V.M. (2016). Effect of Mg^2+^ doping on beta-alpha phase transition in tricalcium phosphate (TCP) bioceramics. Acta Biomater..

[17] Reid J.W., Pietak A., Sayer M., Dunfield D., Smith T.J.N. (2005). Phase formation and evolution in the silicon substituted tricalcium phosphate/apatite system. Biomaterials.

[18] Massie I., Skakle J.M.S., Gibson I.R. (2007). Synthesis and phase stability of silicate-substituted α-tricalcium phosphate. Key Eng. Mater..

[19] Henstock J.R., Canham L.T., Anderson S.I. (2015). Silicon: The evolution of its use in biomaterials. Acta Biomater..

[20] Schwarz K. (1973). A bound form of Si in glycosaminoglycans and polyuronides. Proc. Nat. Acad. Sci. USA.

[21] Carlisle E.M. (1970). Silicon: A possible factor in bone calcification. Science.

[22] Reffitt D.M., Ogston N., Jugdaohsingh R., Cheung H.F.J., Evans B.A.J., Thompson R.P.H., Powell J.J., Hampson G.N. (2003). Orthosilicic acid stimulates collagen type 1 synthesis and osteoblastic differentiation in human osteoblast-like cells in vitro. Bone.

[23] Mladenović Ž., Johansson A., Willman B., Shahabi K., Björn E., Ransjö M. (2014). Soluble silica inhibits osteoclast formation and bone resorption in vitro. Acta Biomater..

[24] Li H., Chang J. (2013). Bioactive silicate materials stimulate angiogenesis in fibroblast and endothelial cell co-culture system through paracrine effect. Acta Biomater..

[25] Gibson I.R., Healy K., Hutmacher D.W., Grainer D.W., Kirkpatrick C.J. (2017). Silicon-containing apatites. Comprehensive Biomaterials II.

[26] Pietak A.M., Reid J.W., Scott M.J., Sayer M. (2007). Silicon substitution in the calcium phosphate bioceramics. Biomaterials.

[27] Bohner M. (2009). Silicon-substituted calcium phosphates—A critical view. Biomaterials.

[28] Szurkowska K., Kolmas J. (2017). Hydroxyapatites enriched in silicon—Bioceramic materials for biomedical and pharmaceutical applications. Prog. Nat. Sci..

[29] Dong G., Zheng Y., He L., Wu G., Deng C. (2016). The effect of silicon doping on the transformation of amorphous calcium phosphate to silicon-substituted α-tricalcium phosphate by heat treatment. Ceram. Int..

[30] Mestres G., le van C., Ginebra M.P. (2012). Silicon-stabilized α-tricalcium phosphate and its use in a calcium phosphate cement: Characterization and cell response. Acta Biomater..

[31] Petrov O.E., Dyulgerova E., Petrov L., Popova R. (2001). Characterization of calcium phosphate phases obtained during the preparation of sintered biphase Ca-P ceramics. Mater. Lett..

[32] Duncan J., Hayakawa S., Osaka A., MacDonald J.F., Hanna J.V., Skakle J.M.S., Gibson I.R. (2014). Furthering the understanding of silicate-substitution in α-tricalcium phosphate: An X-ray diffraction, X-ray fluorescence and solid-state nuclear magnetic resonance study. Acta Biomater..

[33] Serena S., Cabalerro A., de Aza P.N., Sainz M.A. (2015). New evaluation of the in vitro response of silicocarnotite monophasic material. Ceram. Int..

[34] Serena S., Sainz M.A., Cabalerro A. (2014). Single-phase silicocarnotite synthesis in the subsystem Ca_3_PO_4_-Ca_2_SiO_4_. Ceram. Int..

[35] Sayer M., Stratilatov A.D., Reid J., Calderin L., Scott M.J., Yin X., MacKenzie M., Smith T.J.N., Hendry J.A., Langstaff S.D. (2003). Structure and composition of silicon-stabilized tricalcium phosphate. Biomaterials.

[36] Kamitakahara M., Umemoyo S., Ioku K. (2012). Characterization and in vitro evaluation of silicate-containing tricalcium phosphate prepared through wet chemical process. Key Eng. Mater..

[37] Jillavenkatesa A., Condrate R.A. (1998). The infrared and Raman spectra of β- and α-tricalcium phosphate (Ca_3_(PO_4_)_2_). Spectr. Lett..

[38] Berzina-Cimdina L., Borodajenko N., Theophile T. (2012). Research of calcium phosphates using fourier transform infrared spectroscopy. Infrared Spectroscopy—Materials Science, Engineering and Technology.

[39] Hartmann P., Jager C., Barth S., Vogel J., Meyer K. (2001). Solid state NMR, X-ray diffraction and infrared characterization of local structure in heat-treated oxyhydroxyapatite microcrystals: An analog of the thermal decomposition of hydroxyapatite during plasma-spray procedure. J. Solid State Chem..

[40] Kolmas J., Jabloński M., Ślósarczyk A., Kolodziejski W. (2015). Solid-state NMR Study of Mn^2+^ for Ca^2+^ substitution in thermally processed hydroxyapatites. J. Am. Ceram. Soc..

[41] Marchat D., Zymelka M., Cohelo C., Gremillard L., Joly-pottuz L., Babonneau F., Esnouf C., Chevalier J., Bernache-assollant D. (2013). Accurate characterization of pure-silicon-substituted hydroxyapatite powders synthesized by a new precipitation route. Acta Biomater..

[42] Mostafa N.Y., Hassan H.M., Mohamed F.H. (2009). Sintering behavior and thermal stability of Na^+^, SiO_4_^4-^ and CO_3_^2-^ co-substituted hydroxyapatites. J. Alloys Compd..

[43] Galuskin E.V., Galuskina I.O., Gfeller F., Krüger B., Kusz J., Vapnik Y., Dulski M., Dzierżanowski P. (2016). Silicocarnotite, Ca5 [(SiO4)(PO4)](PO4), a new “old’’ mineral from the Negev Desert, Israel, and the ternesite–silicocarnotite solid solution: Indicators of high-temperature alteration of pyrometamorphic rocks of the Hatrurim Complex, Southern Levant. Eur. J. Miner..

[44] Rawal A., Wei X., Akine M., Schmidt-Rohr K. (2008). Dispersion of silicate in tricalcium phosphate elucidated by solid-state NMR. Chem. Mater..

[45] Andreev A.S., Bulina N.V., Chaikina M.V., Prosanov I.Y., Terskikh V.V., Lapina O.B. (2017). Solid-state NMR and computational insights into the crystal structure of silicocarnotite-based bioceramic materials synthesized mechanochemically. Solid State Nucl. Magn. Reson..

[46] Biovia Materials Studio Reflex QPA Datasheet. https://www.3ds.com/fileadmin/PRODUCTS-SERVICES/BIOVIA/PDF/BIOVIA-material-studio-reflex-qpa.pdf.

[47] Crystallography Open Database. http://www.crystallography.net/cod/.

