# Supplementary material for: Effects of Synthesis Conditions on the Formation of Si-Substituted Alpha Tricalcium Phosphates
Source: Int J Mol Sci. 2020 Dec 1;21(23):9164. doi: 10.3390/ijms21239164 (PMC7729989; doi:10.3390/ijms21239164)
Supplement: Supplementary file 1 [file ijms-21-09164-s001.pdf]

Supplementary Materials

Effects of Synthesis Conditions on the Formation of Si-Substituted Alpha Tricalcium Phosphates

Katarzyna Szurkowska [1], Łukasz Szeleszczuk [2] and Joanna Kolmas [1,*]

[1] Chair of Analytical Chemistry and Biomaterials, Department of Analytical Chemistry, Medical University of Warsaw, Faculty of Pharmacy, ul. Banacha 1, 02-097 Warsaw, Poland; katarzyna.szurkowska@wum.edu.pl

[2] Chair of Physical Pharmacy, Department of Physical Chemistry, Medical University of Warsaw, Faculty of Pharmacy, ul. Banacha 1, 02-097 Warsaw, Poland; lukasz.szeleszczuk@wum.edu.pl

* Correspondence: joanna.kolmas@wum.edu.pl; Tel.: +48 22 5720755

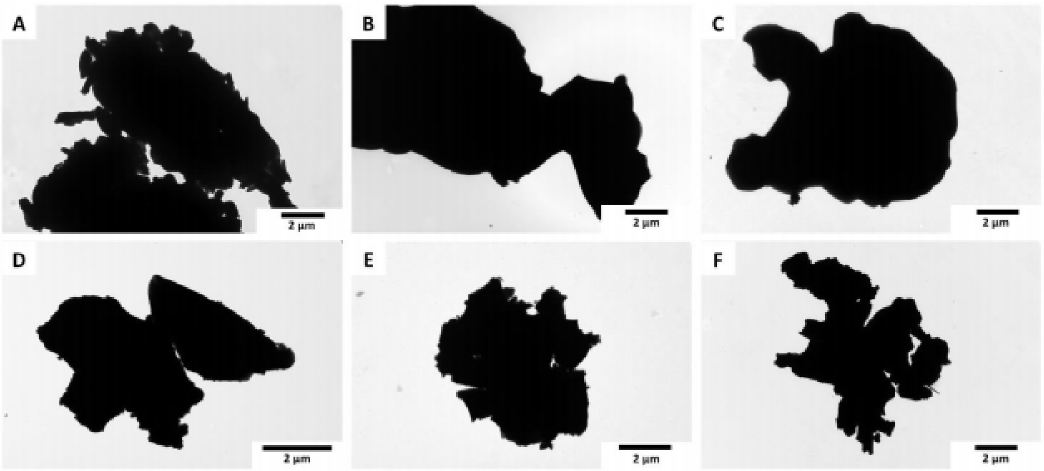

Figure S1. TEM images of 09Si-aTCP (**A**), 18Si-aTCP (**B**), 37-aTCP (**C**), 09Si-aTCPs (**D**), 18Si-aTCPs (**E**) and 37Si-aTCPs (**F**).

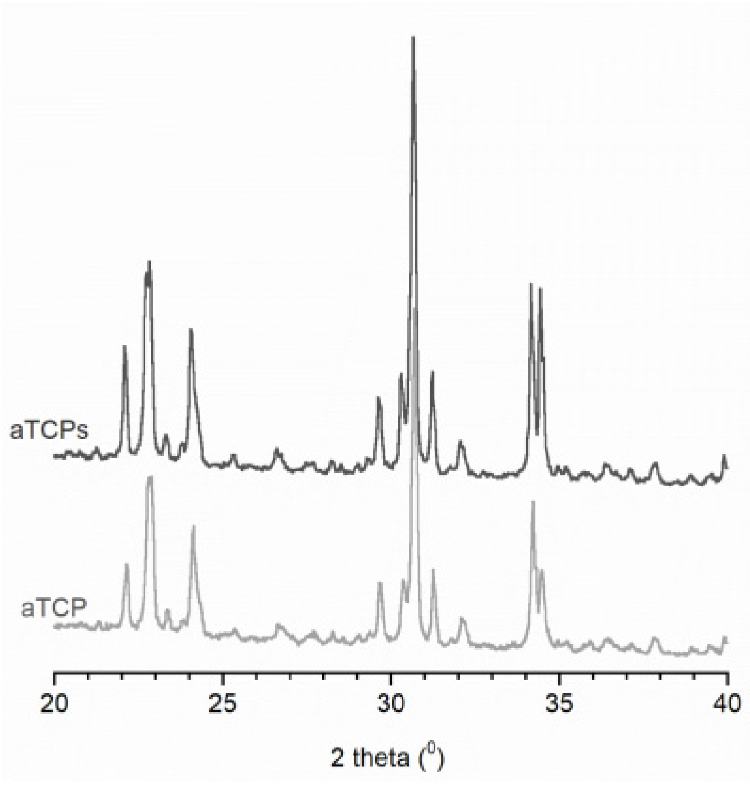

Figure S2. PXRD diffractograms of the aTCP and aTCPs samples.

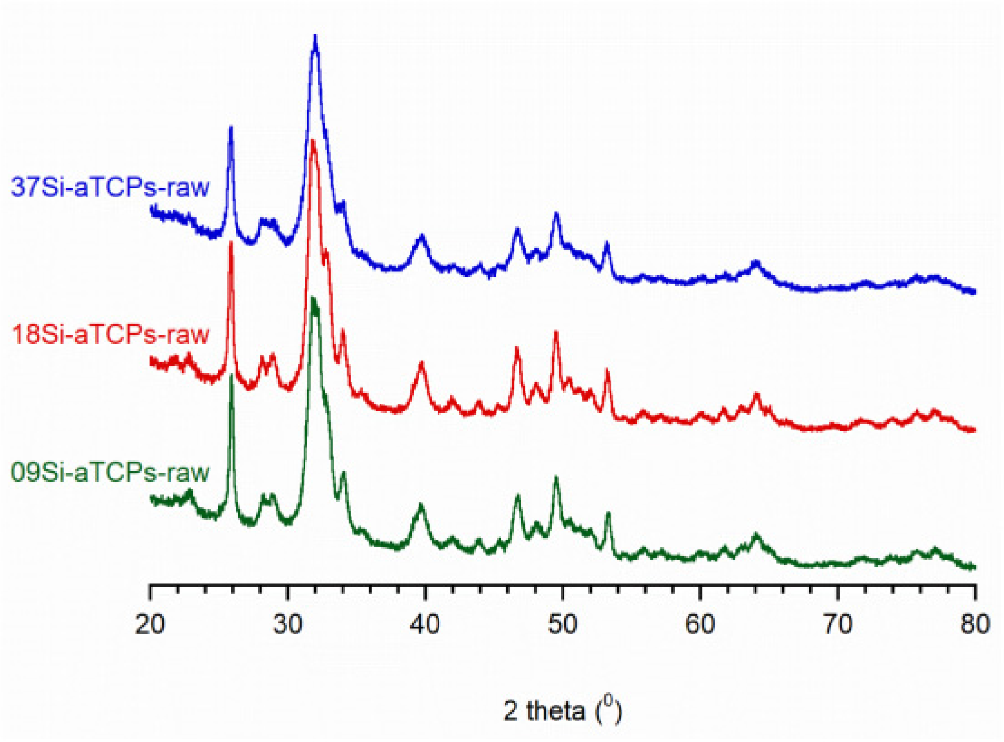

Figure S3: PXRD diffractograms of the raw, unsintered samples obtained by precipitation method.

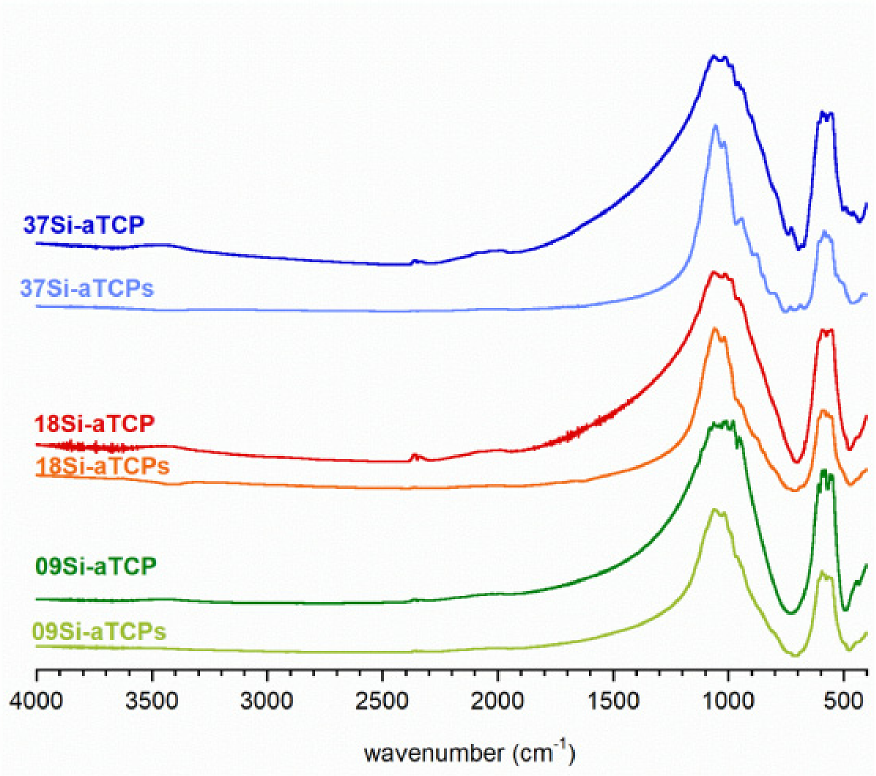

Figure S4. FTIR spectra in 4000-400 cm-1 range.

Table S1: Main peak positions in PXRD diffractograms.

Peak Index	Peak position – 2 theta (°)					
	**09Si-aTCP**	**18Si-aTCP**	**37-aTCP**	**09Si-aTCPs**	**18Si-aTCPs**	**37Si-aTCPs**
**201**	22.20	22.24	22.33	22.28	22.45	22.42
**16-2**	22.84	22.75	22.84	22.80	22.96	22.92
**33-2**	22.92	22.96	23.00	23.00	23.18	23.14
**161**	24.20	24.15	24.20	24.20	24.36	24.28
**36-1**	29.74	29.74	29.83	29.74	29.92	29.87
**40-1**	30.42	30.34	30.38	30.34	30.50	30.46
**034**	30.76	30.80	30.85	30.80	30.97	30.93
**33-5**	31.31	31.31	31.36	31.31	31.48	31.44
**290**	34.28	34.24	34.28	34.24	34.40	34.36
**400**	34.53	34.66	34.75	34.66	34.87	34.78

Table S2. PXRD Data.

Sample	Spacegroup	Parameter *a*	Parameter *b*	Parameter *c*	β
**aTCP**	P21/a	12.8752	27.2760	15.2140	126.190
**aTCPs**	P21/a	12.8828	27.2740	15.2191	126.179
**09Si-aTCPs_raw**	P63/m	9.4310		6.8708	
**18Si-aTCPs_raw**	P63/m	9.4247		6.8756	
**37Si-aTCPs_raw**	P63/m	9.4257		6.8760	
